# Effectiveness of Intensive Primary Care

**DOI:** 10.1007/s11606-018-4409-7

**Published:** 2018-04-09

**Authors:** Anne W. Ekdahl

**Affiliations:** 1 Department of Neurobiology, Care Sciences and Society (NVS), Division of Clinical Geriatrics, Karolinska Institutet, Stockholm, Sweden; 2 Department of Clinical Sciences, Lund University, Helsingborg, Sweden

Dear Editor,

I read with great interest the recent review in JGIM by Dr. Edwards et al. on the “Effectiveness of Intensive Primary Care Interventions.”^1^ Our group published one of the articles included in Dr. Edwards’ review and we are concerned about several discrepancies between our findings and those reported in the review. First, they report that we had 252 patients followed for 2 years. While we experienced losses to follow-up and only included 252 patients in our 2-year analysis of several clinical assessed outcomes, we originally enrolled 382 subjects, and for our 2-year outcomes of death and hospitalization, we included all originally randomized 382 subjects in an intention-to-treat analysis. A second concern is that the article states in Table 2 that we had NR (no report) on the outcomes of hospital admissions and average hospital length of stay. However, we provide extractable data for both outcomes^2^; while we found was no difference between our intervention and control groups for hospital admission rates (2.1 vs. 2.5, *p* = 0.212), our intervention significantly reduced the average length of stay (11.1 vs. 15.2 days, *p* = 0.035). Finally, the review states that they searched through March of 2017 for pertinent articles. However, we published a 3-year follow-up data on our original study cohort in 2016^3^ and in that report, we found that the reduction in average length of stay for hospitalization persisted in our intervention group, the difference in mortality was now significant (27.9%vs. 38.5%, *p* = 0.026), and there was a trend towards a decrease in rates of hospitalization (2.8 vs. 3.4, *p* = 0.06). We are sorry our follow-up study was not included in their systematic review.

I am concerned, saddened actually, that Dr. Edwards’ review lists these “NR” results from the Age-FIT study. I worry that clinicians and policy makers will rely on their presentation of our data and that future readers may misinterpret or be misled about our findings by this article.

Sincerely,

Anne W. Ekdahl, MD, PhD

## References

[1] **Edwards ST, Peterson K, Chan B, Anderson J, Helfand M.** Effectiveness of Intensive Primary Care Interventions: A Systematic Review. Journal of General Internal Medicine. 2017. 10.1007/s11606-017-4174-z PMC5698228 28924747

[2] Ekdahl AW, Wirehn A-B, Alwin J, Jaarsma T, Unosson M, Husberg M (2015). Costs and Effects of an Ambulatory Geriatric Unit (the AGe-FIT Study): A Randomized Controlled Trial. Journal of the American Medical Directors Association..

[3] Ekdahl AW, Alwin J, Eckerblad J, Husberg M, Jaarsma T, Mazya AL (2016). Long-Term Evaluation of the Ambulatory Geriatric Assessment: A Frailty Intervention Trial (AGe-FIT): Clinical Outcomes and Total Costs After 36 Months. Journal of the American Medical Directors Association..

